# Association between Dietary Total Antioxidant Capacity of Antioxidant Vitamins and the Risk of Stroke among US Adults

**DOI:** 10.3390/antiox11112252

**Published:** 2022-11-15

**Authors:** Chaojun Yang, Xiaocan Jia, Yuping Wang, Jingwen Fan, Chenyu Zhao, Yongli Yang, Xuezhong Shi

**Affiliations:** Department of Epidemiology and Biostatistics, College of Public Health, Zhengzhou University, Zhengzhou 450001, China

**Keywords:** total antioxidant capacity, vitamins, oxidative stress, stroke

## Abstract

The intake of antioxidant vitamins can scavenge free radicals and reduce oxidative stress, which may be beneficial for stroke. However, the relationship between total antioxidant capacity (TAC) of antioxidant vitamins and stroke is controversial. This study aims to investigate the association between dietary TAC and the risk of stroke in US adults. This study included participants over 20 years old from the 2001–2018 National Health and Nutrition Examination Survey (NHANES). Data from two 24 h dietary recalls were used to estimate the usual intake of antioxidant vitamins. TAC was calculated by the vitamin C equivalent antioxidant capacity reference values of individual antioxidant vitamins. Survey-weighted generalized linear models were performed to evaluate the relationship between TAC and the risk of stroke. A restricted cubic spline regression model was used to investigate the dose–response association. A total of 37,045 participants was involved, of whom 1391 suffered a stroke. Compared with the first tertile, the participants in the second tertile of TAC showed a lower risk of stroke (OR = 0.788, 95% CI: 0.662, 0.936) after adjusting for potential risk factors. The dose–response analysis showed a gradual increase in the risk of stroke as TAC decreases. Subgroups analyses indicated that this association was primarily in the population of those aged over 60 years old, who were female, consumed alcohol, were a former smoker and inactive. The sensitivity analysis presented consistent results. These results suggest that deficiency of dietary TAC was associated with an increased risk of stroke, particularly in populations with underlying oxidative stress injury.

## 1. Introduction

Stroke has become a global public-health issue. According to the global burden of disease, age-standardized prevalence of stroke diminished by 6.0% from 1990 to 2019, while stroke remained the second-leading cause of death (11.6% of total deaths) and the third-leading cause of death and disability combined (5.7% of total disability adjusted for life years) in 2019 [1]. The well-known risk factors for stroke include hypertension, diabetes, dyslipidemia, cardiovascular disease (CVD), obesity, physical inactivity, smoking and unhealthy diet [2,3]. Thus, the control of these risk factors is crucial for stroke prevention and control.

Healthy lifestyle, such as dietary changes, is encouraged as primary stroke prevention measures [4]. A series of studies showed that dietary intake of antioxidant vitamins was inversely associated with the incidence of stroke [5,6]. This beneficial effect is attributed to antioxidation, which could scavenge free radicals and reduce oxidative stress [7]. The total antioxidant capacity (TAC) is a measure to reflect the antioxidant potential of dietary sources [8]. Some studies exhibited that TAC from the diet is associated with a lower risk of stroke in an Italian cohort [9] and Iranian adults [10]. Meanwhile, two studies of the Swedish population found that dietary antioxidant capacity from different foods and beverages was inversely associated with the risk of stroke in women but not associated in men [11,12], whereas another study showed that higher dietary TAC was not associated with stroke in young and middle-aged Swedish women [13]. Moreover, dietary TAC did not seem to predict risk of stroke for people aged 55 years and older in the Rotterdam study [14]. Therefore, the relationship between TAC and stroke remains unclear and controversial. 

This study aimed to investigate the association between dietary TAC of antioxidant vitamins and the risk of stroke in US adults based on the National Health and Nutrition Examination Survey (NHANES), after adjusting for potential risk factors.

## 2. Materials and Methods

### 2.1. Data Source and Study Population

NHANES is designed to obtain nationally representative information on the health and nutritional status of the US population conducted by the National Center for Health Statistics [15]. This survey is a complex, multistage sampling design, with data collection in their homes and mobile examination center (MEC). The continuous NHANES data are released for public use every 2 years. NHANES study protocols were approved by the NCHS Research Ethics Review Board and received written informed consent from participants [16]. 

**Figure 1:**
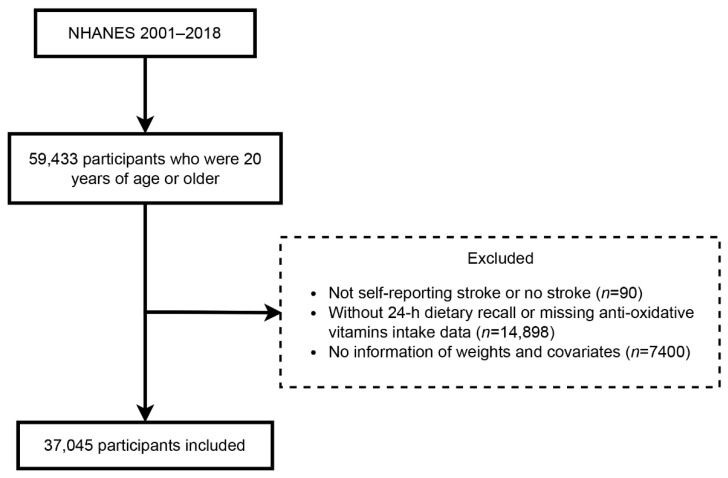
Flowchart for the selection of participants.

We selected participants in NHANES from 2001 to 2018 who were 20 years of age or older (*n* = 59,433). We excluded 22,388 participants (Figure 1): (1) not self-reporting the condition of stroke (*n* = 90); (2) without 24 h dietary recall (DR) or missing antioxidative vitamins intake data (*n* = 14,898); (3) no information of covariables (*n* = 7400). 

### 2.2. Definition of Stroke

The medical conditions questionnaire provided self-reported personal interview data that could be used to obtain the status of stroke. Stroke was defined as a self-report of “a doctor or other health professional ever told you that had a stroke”.

### 2.3. Estimation of TAC from Diet

Dietary data were collected by 2 24 h DRs, including the first day (Day 1) and second day (Day 2) which were collected in via the MEC and telephone, respectively. The in-person interview was conducted in the MEC dietary interview room, reporting amounts of foods by a set of measuring guides (various glasses, bowls, mugs, household spoons, measuring cups and spoons, a ruler, thickness sticks, bean bags and circles). Telephone dietary interviews were self-reporting by telephone using measuring guides 3 to 10 days after the MEC dietary interview. Data of 2 24 h DRs were intended to estimate the usual dietary intake in the US.

The intake of antioxidative vitamins in the NHANES dietary interview consisted of vitamin A, vitamin C, vitamin E, α-carotene, β-carotene, β-cryptoxanthin, lycopene and lutein-zeaxanthin. Floegel et al. [17] developed and validated an algorithm to establish a TAC database of the US diet. This database provided vitamin C equivalent antioxidant capacity reference values of above 8 antioxidant vitamins. TAC of each antioxidative vitamin was calculated by multiplying the individual daily intake of antioxidant vitamin by its antioxidant capacities. Theoretical TAC was determined by summing the TAC of above 8 antioxidative vitamins as following:

$$TAC=\Sigma [Antioxidant\;content\left(\frac{100\;g}{d}\right)\times Antioxidant\;capacity(\frac{mg\;VCE}{100\;g})]$$

*Antioxidant content* represents the daily intake of each antioxidative vitamin, which was collected in NHANES dietary interview. *Antioxidant capacity* is the vitamin C equivalent antioxidant capacity of corresponding antioxidative vitamin, which was derived from TAC database of the US diet [17]. VCE means the equivalent of vitamin C. The estimated TAC was log-transformed or categorized for further analysis. TAC was divided into first tertile (T1: 0 < TAC ≤ 4421.34 mg VCE/d), second tertile (T2: 4421.34 < TAC ≤ 10,100.03 mg VCE/d) and third tertile (T3: 10,100.03 < TAC ≤ 20,0675 mg VCE/d) according to survey-weighted tertiles.

### 2.4. Covariates

Social demography and lifestyle factors were collected by interview and questionnaires.

The sociodemographic factors included age, sex (male and female) and race (non-Hispanic white, non-Hispanic black, Mexican American, non-Hispanic Asian and other race). Lifestyle consisted of alcohol (yes and no), smoking status (never, former and now) and activity (vigorous, moderate and no), which was obtained from questionnaires (Supplementary Table S1).

The risk factors for stroke included diabetes, hypertension, dyslipidemia, CVD, obesity and inflammation [2,3]. The diagnostic criteria of diabetes [18], hypertension [19], dyslipidemia [20] and CVD are shown in Supplementary Table S2. Obesity status was stated by body mass index (BMI) according to World Health Organization [21]: thin (<18.5 kg/m^2^), normal weight (18.5–24.9 kg/m^2^), overweight (25.0–29.9 kg/m^2^) and obesity (≥30.0 kg/m^2^). Neutrophil to lymphocyte ratio (NLR) is an easily accessible inflammatory marker from complete blood count for stroke [22]. NLR was divided into two categories based on median. Above hematological indexes were measured directly from the serum as described in the NHANES laboratory procedures manual.

### 2.5. Statistical Analyses

NHANES data were extracted and preprocessed by R “nhanesR package”. In our study, all analysis was based on sample weights to produce accurate nationwide estimates in US. If the missing data of covariates were less than 10%, the samples with missing data were deleted. The characteristics of sociodemographic, lifestyle and stroke risk factors were described by frequencies (unweighted) and percentages (weighted). The distribution of these characteristics in groups was assessed with Rao–Scott *χ*^2^ tests. The intake of antioxidative vitamins and TAC was reported as medians (P25, P75) and compared between groups based on Mann–Whitney U or Kruskal–Wallis H test.

Survey-weighted generalized linear models were performed to evaluate the relationship between TAC and the risk of stroke, represented by odds ratio (OR) and 95% confidence interval (CI). The relationship between antioxidant vitamins and stroke was also estimated. We fitted three statistical models, of which TAC was log-transformed or categorized by tertiles (T1 as reference). Model 1 was adjusted for age, gender and race. Model 2 was further adjusted for lifestyle factors, including alcohol, smoking and activity. Model 3 included all variables of Model 2 and additionally adjusted for stroke risk factors (diabetes, hypertension, dyslipidemia, CVD, BMI and NLR). ROC curve was used to verify the discrimination of the model. Then, TAC was log-transformed and brought into model 3 as a continuous variable to estimate the dose–response relationship with stroke risk by restricted cubic spline.

The subgroups analyses were conducted by age, gender, smoke, alcohol and activity. Sensitivity analyses were also performed to test the robustness of our findings. First, we exerted multiple interpolation of missing data. Second, we excluded participants who simultaneously have complications of diabetes, hypertension, dyslipidemia and CVD.

All statistical analyses were performed using the R version 4.2.1 software (CRAN team, Vienna, Australia, https://cran.r-project.org/mirrors.html; accessed on 10 September 2022). Two-sided *p* < 0.05 was considered as statistically significant.

## 3. Results

### 3.1. Characteristics of Participants

A total of 37,045 participants, representing 179,355,225, US adults, was included in our study. Among them, 1391 participants (unweighted) suffered from stroke. TAC was lower in the stroke group than that of the no-stroke group (Supplementary Table S3; *p* < 0.05). What‘s more, the proportion of T1 to T3 for TAC decreased gradually in the stroke group. The intake of antioxidant vitamins between participants with stroke and without stroke is shown in Supplementary Table S3. The percentages of elderly (60–85 years old), female, smoking (former and now), no activity, obesity (BMI ≥ 30.0 kg/m^2^), diabetes, hypertension, hyperlipidemia, CVD and NLR ≥ 1.95 were higher in the stroke group than those of participants without stroke (Table 1; *p* < 0.05). In the tertile groups of TAC, T1 had the highest weighted mean rate of stroke with 3.27%, while T2 and T3 were 2.58% and 2.43%, respectively. The distribution of characteristics between groups based on tertiles of TAC is displayed in Supplementary Table S4.

### 3.2. Relationship between TAC and Stroke

**Figure 2:**
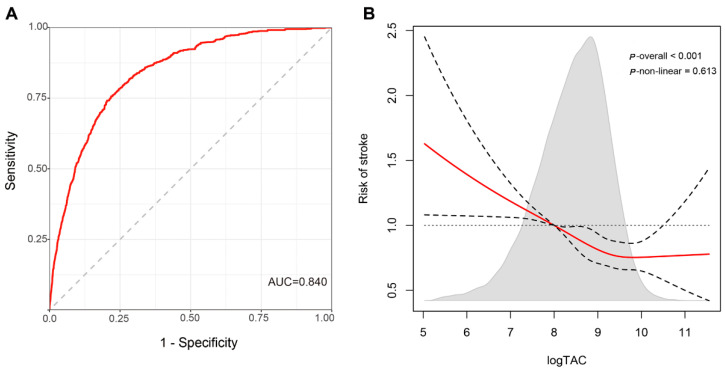
ROC curve and restricted cubic spline plot for the relationship between total antioxidant capacity and the risk of stroke among US adults. (**A**) Survey-weighted ROC curve for the relationship between total antioxidant capacity and the risk of stroke. The ROC curve was adjusted for age, gender, race, alcohol, smoke, activity, diabetes, hypertension, dyslipidemia, CVD, BMI and NLR. CVD: cardiovascular disease; BMI: body mass index; NLR: neutrophil to lymphocyte ratio. (**B**) Restricted cubic spline plot for the relationship between total antioxidant capacity and the risk of stroke. ORs were adjusted for age, gender, race, alcohol, smoke, activity, diabetes, hypertension, dyslipidemia, CVD, BMI and NLR. TAC was log-transformed and the shaded area was the distribution of TAC. A logTAC level of 8 was used as the reference to estimate all ORs. The dashed areas indicate the 95% CI. CVD: cardiovascular disease; BMI: body mass index; NLR: neutrophil to lymphocyte ratio; TAC: total antioxidant capacity.

The ORs and 95% CI for stroke by survey tertiles (T1 to T3) of TAC level are shown in Table 2. In Model 1, lower intake (T1) of TAC showed a higher risk of stroke when compared to higher tertiles of TAC after adjusting for age, sex and race. This association remained identical after further adjustment for lifestyle (alcohol, smoke and activity) in Model 2. After adding adjustment for diabetes, hypertension, hyperlipidemia, CVD, BMI and NLR, the multivariable-adjusted ORs (95% CIs) across tertiles of TAC were 1.00 (reference), 0.788 (0.662, 0.936) and 0.860 (0.726, 1.019). The AUC for this model was 0.840 (Figure 2A). The dose–response analysis showed a gradual increase in the risk of stroke as TAC decreased (*p* < 0.001) (Figure 2B). For the relationship between each antioxidant vitamin and stroke, taking a certain amount of vitamin A, vitamin C, vitamin E, β-carotene, β-cryptoxanthin and lycopene could reduce the risk of stroke, respectively (Supplementary Table S5), whereas there was no relationship between eight antioxidant vitamins and stroke after putting them in the models simultaneously (Supplementary Table S6).

### 3.3. Subgroup Analysis

We further conducted stratified analyses for the associations of TAC with the risk of stroke by age, gender, smoking status, alcohol and activity. In the population of the elderly (more than 60 years old), women, drinkers, former smokers and no activity, inadequate intake (T1) of TAC was associated with a higher risk of stroke (Table 3).

### 3.4. Sensitivity Analysis

In sensitivity analyses, T1 of TAC showed a completely coherent risk of stroke by interpolation of missing data (Table 4). The association of TAC with the risk of stroke was not materially changed when we excluded the participants concurrently with diabetes, hypertension, CVD and dyslipidemia (Supplementary Table S7).

## 4. Discussion

Based on a large sample of nationally representative US adults, we found that deficiency in dietary TAC of antioxidant vitamins was associated with an increased risk of stroke after adjustment for relevant covariates, predominantly in the population of over 60 years, female, drinkers, former smoking and inactivity. The dose–response relationship showed that the risk of stroke increased with the decrease in TAC gradually. Sensitivity analyses demonstrated the robustness of our findings.

The relationship between dietary TAC and stroke in our study is consistent with the previous studies in the population of Italian and Iranian adults [9,10], whereas our results are not consistent with the Swedish study [13] report that there was no association between dietary TAC and stroke. The first reasons for inconsistent results may be racial difference. Hantikainen et al. [13] selected 30- to 49-year-old women who were residing in the Uppsala Health Care region as participants. The second reason is the discrepancy in the collection of dietary information. In our study, 24 h DRs were conducted with a set of measuring guides that could accurately evaluate the usual dietary intake, while Hantikainen et al. [13] used a food frequency questionnaire (FFQ). Third, the method of dietary TAC estimation is different. We calculated TAC as the sum of the product of antioxidant content and antioxidant capacity of antioxidant vitamins. The antioxidant capacity of antioxidant vitamins was derived from references, which was determined by 2,2′-azino-bis (3-ethylbenzthiazoline-6-sulfonic acid (ABTS) assay [17] in our study, ferric-reducing antioxidant power (FRAP) assay [23] in Italian [9], Iranian [10] and Swedish studies [11,12,13]. Fourth, there is dietary pattern discrepancy between countries. A series of research reported that a healthy diet style exerts a beneficial effect on stroke, such as Mediterranean diet [24], DASH diet [25], foods with low inflammatory values [26]. Therefore, the diversity of dietary patterns in different races and genders may affect the association between TAC and the risk of stroke. In general, the discrepancy in ethnicity, type of dietary questionnaire, measurement of TAC and dietary pattern may contribute to the different results.

Our study found that deficiency in dietary TAC was associated with an increased risk of stroke. Oxidative stress and chronic low-grade inflammation share a plethora of common molecular and cellular mechanisms in the pathogenesis of vascular aging and atherosclerosis, involved in atherosclerotic ischemic stroke [27,28]. Oxidative stress is inextricably linked to inflammatory responses, which can initiate inflammatory responses, while it is also a consequence of inflammation [28]. Oxidative stress plays a critical role in the pathophysiology of stroke due to an excessive production of free radicals, overwhelming endogenous antioxidant defense mechanisms and promoting the development of oxidative state and inflammatory response [29]. Afterwards, the increase in inflammatory markers gives rise to endothelial dysfunction, intima thickening and arterial stiffness, leading to aging and damage to blood vessels [30]. Reversely, brain inflammation stimulates oxidative stress, aggravating brain injury by exacerbating blood–brain barrier damage, microvascular failure and brain edema [31]. A series of studies has shown that antioxidants can regulate blood lipids and reduce inflammatory responses, thereby removing oxygen free radicals to protect cerebrovascular and neurons [32,33,34]. Hence, insufficient intake of TAC cannot adequately remove oxygen free radicals and reduce oxidative stress, ultimately resulting in the occurrence and progression of stroke.

Subgroup analysis showed that the association of lower TAC with increased risk of stroke was mainly in the population of over 60 years old, women, drinkers, former smokers and no activity. It is indicated that the effect of the antioxidant diet on stroke is influenced by age, sex and lifestyle. With the increase in age in females, the level of lipid peroxidation levels in follicular fluid gradually increases, accompanied by a change in the antioxidant pattern in vivo that impaired reactive oxygen species (ROS) scavenging efficiency [35]. In postmenopausal women, the extent of serum oxidative stress rose with elevation of serum malondialdehyde and reduction in serum TAC [36]. An experiment revealed that vitamin A was capable of ameliorating antioxidant status in a menopause rat model [37]. High alcohol intake was related to increased oxidative damage and decreased levels of antioxidant enzymes [38]. Meanwhile, Vitamin E could mitigate the oxidative stress induced by alcohol [39]. As is well known, smoking increases oxidative damage and decreases the antioxidant defense system [40]. What’s more, it may take more than 10 years until the redox balance is restored, indicating that tobacco is extraordinarily harmful for the oxidative defense system [41]. Based on antioxidative properties, Vitamin E presented a neuroprotective effect on the impairment of the oxidation defense system induced by waterpipe smoking [42]. Kozakiewicz et al. [43] found that antioxidant enzyme activities were significantly lower in inactive people than those of active ones and physical activity caused a decrease in oxidative stress markers. Therefore, insufficient TAC intake in people who have a certain degree of oxidative stress injury may further aggravate oxidative defense system damage and increase the risk of stroke.

Our study has several strengths. First, we applied TAC as a measurement of the overall antioxidant potential from the diet. In addition, potential confounders were adequately adjusted on the controversial issue of TAC and stroke, particularly in the factors of lifestyle and health status associated with stroke. Furthermore, survey-weighted generalized linear models were used to obtain an unbiased estimation.

This study has some limitations. Firstly, data on stroke and 24 h DR may be subject to recall bias due to self-report. Secondly, as participants only reported “stroke” or “no”, we cannot evaluate the effect of TAC on ischemic stroke or hemorrhagic stroke. Thirdly, the intake of TAC did not include vitamin supplements in our study. Fourth, the bioavailability and in vivo activity of dietary antioxidant vitamins are not considered, because there is only one survey cycle (2003–2004) with complete data of serum antioxidant vitamins. The bioavailability displayed a high variability between individual diets [44], which may affect the effect of dietary TAC on stroke. In the future, we should further investigate the relationship between the TAC in vivo or antioxidative vitamin supplements and the risk of stroke based on cohort study. Then, the underlying biological mechanisms of association between TAC and the risk of stroke could be explored by conducting basic research.

## 5. Conclusions

In conclusion, deficiency of dietary TAC was associated with an increased risk of stroke, especially in the population of potential oxidative stress injury. Our findings support the hypothesis that a physiological dose of antioxidant vitamin diet is beneficial in reducing the risk of stroke.

## Figures and Tables

**Table 1:** Characteristics of the participants between with stroke and without stroke among US adults.

Characteristics	Level	Overall (*n* = 37,045)	No Stroke (*n* = 35,654)	Stroke (*n* = 1391)	*p*
Age (years)	20–39	12,055 (35.73)	11,991 (36.57)	64 (6.29)	<0.001
	40–59	12,266 (38.75)	11,950 (39.05)	316 (28.14)	
	60–85	12,724 (25.52)	11,713 (24.38)	1011 (65.57)	
Sex	Female	18,341 (50.33)	17,654 (50.18)	687 (55.50)	0.003
	Male	18,704 (49.67)	18,000 (49.82)	704 (44.50)	
Race	Non-Hispanic white	17,132 (70.33)	16,389 (70.26)	743 (72.77)	<0.001
	Non-Hispanic black	7434 (10.14)	7082 (10.04)	352 (13.57)	
	Mexican American	6164 (7.98)	6020 (8.08)	144 (4.28)	
	Other Hispanic	3091 (5.14)	3020 (5.21)	71 (2.67)	
	Other race	3224 (6.42)	3143 (6.41)	81 (6.71)	
Smoke	Never	19,895 (53.59)	19,365 (54.00)	530 (39.19)	<0.001
	Former	9358 (25.37)	8832 (25.08)	526 (35.44)	
	Now	7792 (21.04)	7457 (20.91)	335 (25.38)	
Alcohol	No	9756 (21.69)	9317 (21.45)	439 (29.95)	<0.001
	Yes	27,289 (78.31)	26,337 (78.55)	952 (70.05)	
Activity	No	17,773 (41.26)	16,810 (40.56)	963 (65.74)	<0.001
	Moderate	10,072 (29.29)	9729 (29.36)	343 (27.17)	
	Vigorous	9200 (29.45)	9115 (30.08)	85 (7.10)	
BMI (kg/m^2^)		28.84 ± 6.69	28.81 ± 6.68	30.10 ± 6.94	<0.001
	Thin (<18.5)	556 (1.55)	539 (1.56)	17 (1.43)	<0.001
	Normal (18.5–24.9)	10,156 (28.90)	9844 (29.09)	312 (21.98)	
	Overweight (25.0–29.9)	12,548 (33.44)	12,096 (33.50)	452 (31.35)	
	Obesity (≥30.0)	13,785 (36.11)	13,175 (35.85)	610 (45.23)	
Diabetes	No	27,631 (79.58)	26,882 (80.21)	749 (57.59)	<0.001
	Yes	9414 (20.42)	8772 (19.79)	642 (42.41)	
Hypertension	No	20,966 (61.94)	20,715 (63.10)	251 (21.00)	<0.001
	Yes	16,079 (38.06)	14,939 (36.90)	1140 (79.00)	
Hyperlipidemia	No	10,289 (29.01)	10,103 (29.48)	186 (12.45)	<0.001
	Yes	26,756 (70.99)	25,551 (70.52)	1205 (87.55)	
CVD	No	34,276 (94.03)	33,330 (94.76)	946 (68.53)	<0.001
	Yes	2769 (5.97)	2324 (5.24)	445 (31.47)	
NLR	<1.95	19,168 (50.00)	18,613 (50.33)	555 (38.48)	<0.001
	≥1.95	17,877 (50.00)	17,041 (49.67)	836 (61.52)	

Categorical variables were presented as numbers (unweighted) and percentages (weighted). BMI: body mass index; CVD: cardiovascular disease; NLR: neutrophil to lymphocyte ratio.

**Table 2:** Relationship between total antioxidant capacity and the risk of stroke among US adults.

TAC ^a^ (mgVCE/d)	Model 1		Model 2		Model 3	
	OR (95%CI)	*p*	OR (95%CI)	*p*	OR (95%CI)	*p*
T1	1 (reference)		1 (reference)		1 (reference)	
T2	0.666 (0.564, 0.787)	<0.001	0.770 (0.653, 0.908)	0.002	0.788 (0.662, 0.936)	0.007
T3	0.661 (0.562, 0.778)	<0.001	0.836 (0.706, 0.990)	0.038	0.860 (0.726, 1.019)	0.081
logTAC	0.822 (0.771, 0.876)	<0.001	0.913 (0.853, 0.977)	0.009	0.925 (0.864, 0.990)	0.025

Model 1 was adjusted for age, gender and race. Model 2 was Model 1 plus lifestyle factors, including alcohol, smoke and activity. Model 3 was Model 2 plus stroke risk factors, including diabetes, hypertension, dyslipidemia, CVD, BMI and NLR. ^a^ TAC was grouped into T1, T2 and T3 according to tertile (T1 as reference). TAC: total antioxidant capacity; T1: the first tertile; T2: the second tertile; T3: the third tertile; CVD: cardiovascular disease; BMI: body mass index; NLR: neutrophil to lymphocyte ratio.

**Table 3:** Subgroup analysis of relationship between total antioxidant capacity and the risk of stroke among US adults.

Subgroup		T1	T2 ^a^		T3 ^a^	
			OR (95%CI)	*p*	OR (95%CI)	*p*
Age (year)	20–39	1 (reference)	0.520 (0.252, 1.075)	0.077	0.461 (0.201, 1.061)	0.068
	40–59	1 (reference)	0.722 (0.493, 1.057)	0.093	1.006 (0.674, 1.502)	0.977
	60–85	1 (reference)	0.849 (0.686, 0.990)	0.040	0.856 (0.707, 1.035)	0.107
Gender	Female	1 (reference)	0.712 (0.568, 0.892)	0.003	0.875 (0.689, 1.111)	0.270
	Male	1 (reference)	0.931 (0.724, 1.196)	0.572	0.881 (0.675, 1.150)	0.347
Alcohol	No	1 (reference)	0.865 (0.641, 1.167)	0.340	1.078 (0.783, 1.485)	0.642
	Yes	1 (reference)	0.756 (0.603, 0.948)	0.016	0.782 (0.624, 0.979)	0.032
Smoke	Never	1 (reference)	0.861 (0.653, 1.134)	0.283	1.002 (0.764, 1.313)	0.990
	Former	1 (reference)	0.700 (0.538, 0.911)	0.008	0.732 (0.539, 0.992)	0.044
	Now	1 (reference)	0.820 (0.580, 1.161)	0.261	0.834 (0.561, 1.241)	0.367
Activity	No	1 (reference)	0.738 (0.614, 0.887)	0.001	0.890 (0.729, 1.087)	0.250
	Moderate	1 (reference)	0.864 (0.593, 1.259)	0.444	1.014 (0.711, 1.447)	0.936
	Vigorous	1 (reference)	0.835 (0.430, 1.619)	0.590	0.818 (0.734, 1.053)	0.052

^a^ TAC was grouped into T1, T2 and T3 according to tertile (T1 as reference). The models were adjusted for age, gender, race, alcohol, smoke, activity, diabetes, hypertension, dyslipidemia, CVD, BMI and NLR. The strata variable was not included in the model when stratifying by itself. TAC: total antioxidant capacity; T1: the first tertile; T2: the second tertile; T3: the third tertile; CVD: cardiovascular disease; BMI: body mass index; NLR: neutrophil to lymphocyte ratio.

**Table 4:** Sensitivity analysis of relationship between total antioxidant capacity and the risk of stroke by interpolation of missing data ^a^.

TAC ^b^ (mgVCE/d)	Model 1		Model 2		Model 3	
	OR (95% CI)	*p*	OR (95% CI)	*p*	OR (95% CI)	*p*
T1	1 (reference)		1 (reference)		1 (reference)	
T2	0.660 (0.569, 0.765)	<0.001	0.750 (0.648, 0.868)	<0.001	0.768 (0.660, 0.892)	<0.001
T3	0.661 (0.573, 0.762)	<0.001	0.816 (0.706, 0.944)	0.007	0.837 (0.721, 1.002)	0.060
logTAC	0.822 (0.777, 0.870)	<0.001	0.901 (0.849, 0.957)	<0.001	0.911 (0.858, 0.967)	0.003

Model 1 was adjusted for age, gender and race; Model 2 was Model 1 plus lifestyle factors, including alcohol, smoke and activity; Model 3 was Model 2 plus stroke risk factors, including diabetes, hypertension, dyslipidemia, CVD, BMI and NLR. ^a^ After interpolation of missing data, there were 44,445 participants in this sensitivity. ^b^ TAC was grouped into T1, T2 and T3 according to tertile (T1 as reference). TAC: total antioxidant capacity; T1: the first tertile; T2: the second tertile; T3: the third tertile; CVD: cardiovascular disease; BMI: body mass index; NLR: neutrophil to lymphocyte ratio.

## Data Availability

The data were publicly available from NHANSE (https://www.cdc.gov/nchs/nhanes/; accessed on 11 June 2022). All data generated or analyzed during this study are included in this published article.

## Supplementary Materials

The following supporting information can be downloaded at: https://www.mdpi.com/article/10.3390/antiox11112252/s1, Table S1: Questionnaires and classifications of lifestyle factors; Table S2: Diagnostic criteria of diabetes, hypertension, dyslipidemia and CVD in this study; Table S3: Intake of antioxidant vitamins between participants with stroke and without stroke; Table S4: Characteristics of the participants by weighted tertile of total antioxidant capacity among US adults; Table S5: Relationship between each antioxidant vitamin and stroke; Table S6: Relationship between eight antioxidant vitamins and stroke; Table S7: Sensitivity analysis of relationship between total antioxidant capacity and risk of stroke after exclusion of participants with complications.
