# Supplementary material for: Association between Dietary Total Antioxidant Capacity of Antioxidant Vitamins and the Risk of Stroke among US Adults
Source: Antioxidants (Basel). 2022 Nov 15;11(11):2252. doi: 10.3390/antiox11112252 (PMC9686933; doi:10.3390/antiox11112252)
Supplement: Supplementary file 1 [file antioxidants-11-02252-s001.zip › antioxidants-1965264-supplementary.pdf]

Table S1 Questionnaires and classifications of lifestyle factors

Lifestyle factor	Questionnaire	Classification
**Smoke**	Smoking and tobacco use questionnaire	• **Never**：smoked less than 100 cigarettes in life<br>• **Former**：smoked at least 100 cigarettes in life and not at all now<br>• **Now**：smoked at least 100 cigarettes in life and smoke now
**Alcohol**	Alcohol use questionnaire	• **No**：have not at least 12 drinks of any type of alcoholic beverage<br>• **Yes**：have at least 12 drinks of any type of alcoholic beverage
**Activity**	Physical activity questionnaire or global physical activity questionnaire	• **No**：no physical activity<br>• **Moderate**：do moderate activities that cause only light sweating or a slight to moderate increase in breathing or heart rate<br>• **Vigorous**：do any vigorous activities that cause heavy sweating, or large increases in breathing or heart rate

Table S2 Diagnostic criteria of diabetes, hypertension, dyslipidemia and CVD in this study

Disease	Diagnostic criteria
**Diabetes**	ADA guideline[1]:<br>• HbA1c ⩾ 6.5%<br>• Random blood sugar⩾ 11.1mmol/L<br>• Oral glucose tolerance test 2 h blood glucose⩾11.1mmol/L<br>• Fasting blood-glucose⩾7.0 mmol/L<br>• Self-report of diabetes (“doctor told you have diabetes”) or treatment for diabetes (“taking insulin now” or “taking diabetic pills to lower blood sugar”)
**Hypertension**	JNC7 guideline[2]:<br>• Average blood pressure≥ 140/90 mmHg [a]<br>• Self-report of hypertension (“doctor told you have hypertension”) or treatment for hypertension (“taking prescription for hypertension”)
**Dyslipidemia**	Dyslipidemia Management in 2020[3]:<br>• Triglyceride⩾150 mg/dL<br>• Total cholesterol⩾200 mg/dL<br>• High density lipoprotein cholesterol <40 mg/dL in male or 50 mg/dL in female<br>• Low density lipoprotein cholesterol⩾130 mg/dL

	• Drug treatment for regulation of blood lipids
**CVD**	Self-report of congestive heart failure or coronary heart disease or angina

[a] After resting quietly in a sitting position for 5 minutes, three consecutive blood pressure readings were taken to calculate the average value.

Table S3 Intake of antioxidant vitamins between participants with stroke and without stroke

Vitamin [a]	Level	Overall (n=37045)	No stroke (n=35654)	Stroke (n=1391)	*P*
**TAC (mg VCE/d)**		6805.30[3435.14, 12194.66]	6836.12[3448.83, 12221.39]	5785.95[3047.23, 11107.94]	<0.001
	**T1**	12088(33.33)	11560(33.16)	528(39.47)	0.001
	**T2**	12219(33.33)	11764(33.40)	455(31.18)	
	**T3**	12738(33.33)	12330(33.45)	408(29.35)	
**Vitamin A (mcg/d)**		529.00[325.00, 814.00]	530.00[325.50, 815.50]	486.36[292.14, 752.63]	0.001
	**T1**	13686(33.33)	13128(33.24)	558(38.26)	0.012
	**T2**	12154(33.33)	11692(33.32)	462(32.35)	
	**T3**	11205(33.33)	10834(33.44)	371(29.39)	
**Vitamin C (mg/d)**		60.90[28.60, 113.80]	61.15[28.70, 113.95]	52.23[25.11, 105.58]	<0.001
	**T1**	12020(33.33)	11496(33.23)	524(39.78)	<0.001
	**T2**	12182(33.33)	11732(33.35)	450(30.42)	
	**T3**	12843(33.33)	12426(33.42)	417(29.80)	
**Vitamin E (mg/d)**		7.00[4.76, 10.15]	7.03[4.78, 10.20]	6.05[4.04, 8.58]	<0.001
	**T1**	14041(33.33)	13365(33.03)	676(44.67)	<0.001
	**T2**	12175(33.33)	11754(33.41)	421(31.35)	
	**T3**	10829(33.33)	10535(33.56)	294(23.98)	
**α-carotene (mcg/d)**		74.00[22.50, 423.00]	74.50[22.50, 425.00]	69.63[18.00, 347.50]	0.053
	**T1**	12318(33.33)	11810(33.28)	508(35.38)	0.421
	**T2**	12442(33.33)	11996(33.39)	446(33.04)	
	**T3**	12285(33.33)	11848(33.33)	437(31.58)	
**β-carotene (mcg/d)**		1068.00[432.00, 2713.00]	1074.00[434.50, 2725.00]	896.47[346.78, 2367.06]	<0.001
	**T1**	12933(33.33)	12386(33.20)	547(38.30)	0.008
	**T2**	12130(33.33)	11697(33.38)	433(31.89)	
	**T3**	11982(33.33)	11571(33.43)	411(29.81)	
**β-cryptoxanthin (mcg/d)**		39.00[12.00, 110.00]	39.00[12.50, 110.00]	38.50[10.90, 111.33]	0.352
	**T1**	12065(33.33)	11582(33.53)	483(35.19)	0.111
	**T2**	12129(33.33)	11697(33.28)	432(30.05)	
	**T3**	12851(33.33)	12375(33.19)	476(34.76)	
**Lycopene (mcg/d)**		2649.00[660.00, 7161.55]	2677.22[685.00, 7191.00]	1610.45[193.30, 6228.91]	<0.001
	**T1**	13295(33.33)	12645(33.04)	650(43.96)	<0.001
	**T2**	12250(33.33)	11857(33.46)	393(28.70)	
	**T3**	11500(33.33)	11152(33.50)	348(27.35)	

**Lutein zeaxanthin (mcg/d)**		818.50[437.00, 1616.61]	822.00[439.00, 1625.00]	717.14[391.38, 1314.28]	<0.001
	**T1**	12876(33.33)	12336(33.22)	540(38.79)	<0.001
	**T2**	12591(33.33)	12104(33.26)	487(34.56)	
	**T3**	11578(33.33)	11214(33.52)	364(26.65)	

[a] Each vitamin and TAC were grouped into T1, T2 and T3 according to tertile (T1 as reference). Categorical variables were presented as numbers (unweighted) and percentages (weighted). Continuous variables were reported by weighted medians (P25, P75). T1: the first tertile; T2: the second tertile; T3: the third tertile.

Table S4 Characteristics of the participants by weighted tertile of total antioxidant capacity among US adults

**Characteristics**	**Level**	**Total population**	**TAC (mg VCE/d) [a]**			
		**(n=37045)**	**T1(n=12088)**	**T2(n=12219)**	**T3(n=12738)**	***P***
**Stroke**	**No**	35654(97.24)	11560(96.73)	11764(97.42)	12330(97.57)	0.001
	**Yes**	1391(2.76)	528(3.27)	455(2.58)	408(2.43)	
**Age (year)**	**20-39**	12055(35.73)	4166(38.50)	3721(33.48)	4168(35.21)	<0.001
	**40-59**	12266(38.75)	4118(39.18)	4045(39.42)	4103(37.66)	
	**60~85**	12724(25.52)	3804(22.32)	4453(27.10)	4467(27.13)	
**Sex**	**Female**	18341(50.33)	6136(51.97)	6320(52.47)	5885(46.56)	<0.001
	**Male**	18704(49.67)	5952(48.03)	5899(47.53)	6853(53.44)	
**Race**	**Non-Hispanic white**	17132(70.33)	5985(72.41)	5687(71.30)	5460(67.26)	<0.001
	**Non-Hispanic black**	7434(10.14)	2482(10.24)	2301(9.23)	2651(10.95)	
	**Mexican American**	6164(7.98)	1831(7.02)	2077(7.86)	2256(9.05)	
	**Other Hispanic**	3091(5.14)	922(4.67)	1022(5.01)	1147(5.73)	
	**Other race**	3224(6.42)	868(5.65)	1132(6.60)	1224(7.00)	
**Smoke**	**Never**	19895(53.59)	5678(46.80)	6693(54.94)	7524(59.04)	<0.001
	**Former**	9358(25.37)	2833(23.08)	3310(27.09)	3215(25.94)	
	**Now**	7792(21.04)	3577(30.12)	2216(17.97)	1999(15.02)	
**Alcohol**	**No**	9756(21.69)	3129(21.23)	3269(22.00)	3358(21.83)	0.488
	**Yes**	27289(78.31)	8959(78.77)	8950(78.00)	9380(78.17)	
**Activity**	**No**	17773(41.26)	6717(49.31)	5761(40.04)	5295(34.42)	<0.001
	**Moderate**	10072(29.29)	2970(27.31)	3538(31.30)	3564(29.28)	
	**Vigorous**	9200(29.45)	2401(23.38)	2920(28.67)	3879(36.30)	
**BMI (kg/m^2)**	**Normal (18.5-24.9)**	10156(28.90)	3099(25.81)	3322(29.04)	3735(31.85)	<0.001
	**Thin (<18.5)**	556(1.55)	221(1.83)	154(1.27)	181(1.56)	
	**Overweight (25.0-29.9)**	12548(33.44)	3920(32.17)	4145(33.61)	4483(34.54)	
	**Obesity (≥30.0)**	13785(36.11)	4848(40.20)	4598(36.09)	4339(32.05)	
**Diabetes**	**No**	27631(79.58)	8893(78.69)	8999(78.88)	9739(81.18)	0.001
	**Yes**	9414(20.42)	3195(21.31)	3220(21.12)	2999(18.82)	
**Hypertension**	**No**	20966(61.94)	6779(61.77)	6857(62.13)	7330(61.91)	0.911
	**Yes**	16079(38.06)	5309(38.23)	5362(37.87)	5408(38.09)	
**Hyperlipidemia**	**No**	10289(29.01)	3188(26.89)	3438(29.98)	3663(30.15)	<0.001

	Yes	26756(70.99)	8900(73.11)	8781(70.02)	9075(69.85)	
CVD	No	34276(94.03)	11148(93.88)	11306(94.04)	11822(94.18)	0.693
	Yes	2769(5.97)	940(6.12)	913(5.96)	916(5.82)	
NLR	<1.95	19168(50.00)	6093(48.91)	6382(50.50)	6693(50.59)	0.085
	≥1.95	17877(50.00)	5995(51.09)	5837(49.50)	6045(49.41)	

[a] TAC was grouped into T1, T2 and T3 according to tertile (T1 as reference). Categorical variables were presented as numbers(unweighted) and percentages(weighted). TAC: total antioxidant capacity; BMI: body mass index; CVD: cardiovascular disease; NLR: neutrophil to lymphocyte ratio; T1: the first tertile; T2: the second tertile; T3: the third tertile.

Table S5 Relationship between each antioxidant vitamin and stroke

Vitamin [a]		Model 1		Model 2		Model 3	
		OR (95% CI)	*P*	OR (95% CI)	*P*	OR (95% CI)	*P*
**Vitamin A**	**T1**	1(reference)		1(reference)		1(reference)	
	**T2**	0.722(0.595, 0.877)	0.001	0.806(0.663, 0.981)	0.031	0.808(0.665, 0.983)	0.034
	**T3**	0.655(0.547, 0.785)	<0.001	0.787(0.654, 0.948)	0.012	0.823(0.681, 0.995)	0.045
**Vitamin C**	**T1**	1(reference)		1(reference)		1(reference)	
	**T2**	0.652(0.553, 0.769)	<0.001	0.756(0.642, 0.889)	<0.001	0.759(0.640, 0.901)	0.002
	**T3**	0.645(0.549, 0.758)	<0.001	0.823(0.697, 0.972)	0.022	0.832(0.704, 0.982)	0.030
**Vitamin E**	**T1**	1(reference)		1(reference)		1(reference)	
	**T2**	0.714(0.598, 0.851)	<0.001	0.785(0.658, 0.938)	0.008	0.842(0.704, 1.008)	0.061
	**T3**	0.581(0.492, 0.685)	<0.001	0.690(0.585, 0.813)	<0.001	0.751(0.633, 0.890)	0.001
**α-carotene**	**T1**	1(reference)		1(reference)		1(reference)	
	**T2**	0.774(0.649, 0.922)	0.005	0.883(0.740, 1.054)	0.167	0.874(0.728, 1.050)	0.149
	**T3**	0.669(0.564, 0.792)	<0.001	0.809(0.680, 0.962)	0.017	0.809(0.677, 0.967)	0.020
**β-carotene**	**T1**	1(reference)		1(reference)		1(reference)	
	**T2**	0.712(0.596, 0.850)	<0.001	0.797(0.667, 0.953)	0.013	0.819(0.683, 0.982)	0.032
	**T3**	0.586(0.498, 0.690)	<0.001	0.738(0.626, 0.869)	<0.001	0.772(0.653, 0.912)	0.003
**β-cryptoxanthin**	**T1**	1(reference)		1(reference)		1(reference)	
	**T2**	0.763(0.645, 0.903)	0.002	0.837(0.707, 0.990)	0.038	0.831(0.699, 0.987)	0.036
	**T3**	0.803(0.693, 0.931)	0.004	0.955(0.818, 1.114)	0.553	0.935(0.798, 1.094)	0.398
**Lycopene**	**T1**	1(reference)		1(reference)		1(reference)	
	**T2**	0.751(0.628, 0.897)	0.002	0.799(0.668, 0.957)	0.015	0.820(0.682, 0.985)	0.034
	**T3**	0.791(0.664, 0.943)	0.009	0.850(0.713, 1.014)	0.070	0.864(0.722, 1.033)	0.108
**Lutein zeaxanthin**	**T1**	1(reference)		1(reference)		1(reference)	
	**T2**	0.768(0.646, 0.914)	0.003	0.844(0.710, 1.003)	0.053	0.847(0.713, 1.007)	0.059
	**T3**	0.556(0.468, 0.660)	<0.001	0.694(0.583, 0.825)	<0.001	0.736(0.619, 0.875)	<0.001

[a] Each vitamin was grouped into T1, T2 and T3 according to tertile (T1 as reference).

Model 1 was adjusted for age, gender and race;

Model 2 was Model 1 plus lifestyle factors, including alcohol, smoking and activity;

Model 3 was Model 2 plus stroke risk factors, including diabetes, hypertension, dyslipidemia and CVD, BMI and NLR.

T1: the first tertile; T2: the second tertile; T3: the third tertile; CVD: cardiovascular disease; BMI: body mass index; NLR: neutrophil to lymphocyte ratio.

Table S6 Relationship between eight antioxidant vitamins and stroke

Vitamin [a]		Model 1		Model 2		Model 3	
		OR (95% CI)	*P*	OR (95% CI)	*P*	OR (95% CI)	*P*
**Vitamin A**	**T1**	1(reference)		1(reference)		1(reference)	
	**T2**	0.908(0.727, 1.136)	0.396	0.927(0.739, 1.162)	0.508	0.912(0.725, 1.146)	0.424
	**T3**	0.968(0.752, 1.247)	0.801	0.992(0.768, 1.281)	0.952	1.004(0.771, 1.308)	0.975
**Vitamin C**	**T1**	1(reference)		1(reference)		1(reference)	
	**T2**	0.771(0.633, 0.940)	0.011	0.833(0.685, 1.012)	0.065	0.829(0.678, 1.014)	0.067
	**T3**	0.812(0.643, 1.027)	0.081	0.922(0.730, 1.164)	0.489	0.917(0.725, 1.159)	0.464
**Vitamin E**	**T1**	1(reference)		1(reference)		1(reference)	
	**T2**	0.831(0.693, 0.997)	0.047	0.855(0.711, 1.028)	0.095	0.916(0.759, 1.105)	0.356
	**T3**	0.735(0.608, 0.889)	0.002	0.778(0.643, 0.941)	0.010	0.843(0.691, 1.030)	0.094
**α-carotene**	**T1**	1(reference)		1(reference)		1(reference)	
	**T2**	0.968(0.805, 1.164)	0.725	1.011(0.840, 1.217)	0.906	0.978(0.807, 1.184)	0.815
	**T3**	0.932(0.751, 1.157)	0.521	0.979(0.788, 1.216)	0.847	0.942(0.756, 1.175)	0.593
**β-carotene**	**T1**	1(reference)		1(reference)		1(reference)	
	**T2**	0.908(0.746, 1.104)	0.329	0.923(0.759, 1.121)	0.415	0.951(0.778, 1.162)	0.620
	**T3**	0.878(0.676, 1.140)	0.326	0.932(0.721, 1.205)	0.588	0.960(0.739, 1.247)	0.758
**β-cryptoxanthin**	**T1**	1(reference)		1(reference)		1(reference)	
	**T2**	0.948(0.786, 1.144)	0.575	0.966(0.800, 1.168)	0.721	0.951(0.780, 1.160)	0.616
	**T3**	1.115(0.900, 1.383)	0.317	1.153(0.928, 1.432)	0.196	1.108(0.883, 1.390)	0.372
**Lycopene**	**T1**	1(reference)		1(reference)		1(reference)	
	**T2**	0.824(0.685, 0.990)	0.039	0.841(0.698, 1.012)	0.067	0.863(0.715, 1.042)	0.125
	**T3**	0.960(0.794, 1.162)	0.673	0.956(0.788, 1.161)	0.650	0.950(0.780, 1.158)	0.611
**Lutein zeaxanthin**	**T1**	1(reference)		1(reference)		1(reference)	
	**T2**	0.924(0.763, 1.119)	0.414	0.929(0.769, 1.122)	0.441	0.928(0.769, 1.119)	0.429
	**T3**	0.755(0.604, 0.942)	0.013	0.806(0.648, 1.002)	0.052	0.840(0.670, 1.053)	0.128

[a] Each vitamin was grouped into T1, T2 and T3 according to trertiles (T1 as reference).

Model 1 was adjusted for age, gender, race and other seven vitamins;

Model 2 was Model 1 plus lifestyle factors, including alcohol, smoking and activity;

Model 3 was Model 2 plus stroke risk factors, including diabetes, hypertension, dyslipidemia and CVD, BMI and NLR.

T1: the first tertile; T2: the second tertile; T3: the third tertile; CVD: cardiovascular disease; BMI:

body mass index; NLR: neutrophil to lymphocyte ratio.

Table S7 Sensitivity analysis of relationship between total antioxidant capacity and risk of stroke after exclusion of participants with complications [a]

TAC [b] (mg VCE/d)	Model 1		Model 2		Model 3	
	OR (95% CI)	*P*	OR (95% CI)	*P*	OR (95% CI)	*P*
**T1**	1(reference)		1(reference)		1(reference)	
**T2**	0.671(0.556,0.808)	<0.001	0.773(0.643,0.929)	0.007	0.789(0.653,0.954)	0.015
**T3**	0.647(0.543,0.771)	<0.001	0.818(0.681,0.982)	0.031	0.819(0.680,0.985)	0.034
**LogTAC**	0.812(0.759,0.868)	<0.001	0.900(0.839,0.967)	0.004	0.903(0.841,0.970)	0.006

[a] After exclusion of individuals with diabetes, hypertension, CVD and dyslipidemia, simultaneously, there were 36,104 participants in this sensitivity.

[b] TAC was grouped into T1, T2 and T3 according to terrtiles (T1 as reference).

Model 1 was adjusted for age, gender and race;

Model 2 was Model 1 plus lifestyle factors, including alcohol, smoking and activity;

Model 3 was Model 2 plus stroke risk factors, including diabetes, hypertension, dyslipidemia, cardiovascular disease, BMI and NLR.

TAC: total antioxidant capacity; T1: the first tertile; T2: the second tertile; T3: the third tertile; CVD: cardiovascular disease; BMI: body mass index; NLR: neutrophil to lymphocyte ratio.

References:

1. 2. Classification and Diagnosis of Diabetes: Standards of Medical Care in Diabetes-2021. *Diabetes Care* **2021**, 44, S15-S33.

2. Chobanian, A. V.; Bakris, G. L.; Black, H. R.; Cushman, W. C.; Green, L. A.; Izzo, J. J.; Jones, D. W.; Materson, B. J.; Oparil, S.; Wright, J. J.; Roccella, E. J. Seventh report of the Joint National Committee on Prevention, Detection, Evaluation, and Treatment of High Blood Pressure. *Hypertension* **2003**, 42, 1206-52.

3. Karantas, I. D.; Okur, M. E.; Okur, N. U.; Siafaka, P. I. Dyslipidemia Management in 2020: An Update on Diagnosis and Therapeutic Perspectives. *Endocr Metab Immune Disord Drug Targets* **2021**, 21, 815-834.
